# First as a Tragedy, Then as some Other Figure: On Tropology of History and Politics of Literature

**DOI:** 10.12688/openreseurope.20776.1

**Published:** 2025-07-25

**Authors:** Zvonimir Glavaš

**Affiliations:** 1 University of Zagreb, Faculty of Humanities and Social Sciences, Zagreb, 10000, Croatia

**Keywords:** Hayden White, tropology of history, literariness of history, politics of history, politics of literature

## Abstract

This paper explores the intersection of historical representation and literary theory through the lens of tropology, examining how narrative forms shape the political and epistemological dimensions of historiography and how different approaches to the politics of literature within literary theory can help consider the political and ethical implications of historical discourse. Drawing primarily on Hayden White’s notion of the tropological structure of historical discourse, this paper situates his arguments alongside post-foundational political theory, particularly Ernesto Laclau’s and Jacques Rancière’s theoretical frameworks. The paper further examines the applicability of various conceptions of politics of literature in the historiographical field, together with the implications of adopting various modernist literary strategies in historiography, assessing their capacity to challenge realist conventions and amplify marginalized voices. While recognizing the emancipatory potential of such transfers, the paper also critiques their romanticization, cautioning against the uncritical collapse of disciplinary boundaries. Ultimately, it advocates a nuanced engagement with literary theory to enrich historical practice without undermining the specific institutional and epistemological frameworks that differentiate the two fields.

## I.

Hegel says somewhere that great historic facts and personages recur twice. He forgot to add: ‘Once as tragedy, and again as farce.’” ( 1 on page 1) These are, of course, the opening lines of Marx’s *The Eighteenth Brumaire of Louis Bonaparte*. With reference to this, Hayden White asks in *The Content of the Form* ( 2 on page 46) whether there is any logical or empirical test by which the truth value of this assertion can be determined. The factual accuracy of the events of 1851 is certainly testable, but is there a set of objective criteria by which a particular chain of events is to be considered a tragedy or a farce (or a revolution, coup d'état, or whatever)? The question is rhetorical and serves White as one of many arguments to show that what distinguishes narrative history from other representations of past events, such as annals or chronicles, is the possession of “a "structure, an order of meaning” ( 2 on page 5) that it assigns to a group of events using one of the possible models of representation.

A concept originally developed in his seminal work *Metahistory* ( 3), in which White explores the “deep structure of the historical imagination of nineteenth-century Europe” (White 3 on page 2), that is, structures of emplotment, argumentation, and ideological implications underlying historical representations, is further developed in the essays of *The Content of Form* ( 2) and *Figural Realism* ( 4) ^1^. In one of the first essays, White argues that historical discourse, unlike annals and their vertical listing of events, forms “chains of semantic metonymies that would transform his list of events into a discourse about the events considered as a totality evolving in time.” ( 2 on page 16) According to him, this process of assigning a meaning that is in no way inherent in the events themselves, analogous to the way myths or literature function, is a kind of *allegoresis*, which is why “we should regard [any historical narrative] as allegorical, that is, as saying one thing and meaning another.” ( 2 on page 45)

The allegoresis in question, the construction of metonymic chains, requires what White calls a common “metaphysical principle by which to translate difference into similarity” ( 2 on page 16), essentially a center of structure. In seeking the origin of such a principle, he draws on Hegel’s *Philosophy of History* and its claim that “a specifically historical mode of being was linked to a specifically narrative mode of representation by a shared ‘internal vital principle’”; which is “nothing other than politics, which was both the precondition of the kind of interest in the past that informed historical consciousness and the pragmatic basis for the production and preservation of the kinds of records that made historical inquiry possible.” ( 2 on page 27)

Ironically, as White ( 2 on page 61) argues, traditional historical discourse gained its prestige precisely in opposition to various philosophies of history (of which Hegel’s was most influential for a long period of time), contrasting its supposed objectivity and empiricism with the metaphysical nature and ideological undertone of their grand narratives and becoming the privileged ground for judging their concepts. The central political aspect of such a practice was to oppose allegedly disciplined historical consciousness to “utopian thinking in all its forms” ( 2 on page 61), that is, to discourage and dismiss any radical rethinking of social ethos with a sober narrative that relied on it and empowered it. ^2^ But already in one of the basic conclusions of his *Metahistory*, White emphasized that “there can be no ‘proper history’ which is not at the same time ‘philosophy of history’” ( 3 on page xii). Since this means that any historical account, despite its feigned objectivity, is necessarily structured by its ideological judgment of phenomena, which is noticeable in the modes of emplotment and (especially) the figures used, one can conclude that “if there is any logic presiding over the transition from the level of fact or event in the discourse to that of narrative, it is the logic of figuration itself, which is to say, tropology.” ( 2 on page 47) Indeed, White ( 2 on page 149), citing Jameson, has shown that the significance of the tropological is not only to ascribe a particular meaning to events (e.g. a tragedy or a farce), but also to shape a particular causality in the history that is distinct from teleological, mechanical and structural causality.

Yet despite the prominence it brought him, White’s tropological perspective was poorly received by a considerable number of professional historians, who criticized him for succumbing to the infamous “pan-textualism” ( 6 on page 481). The cardinal sin of his alleged relativism was twofold: by exposing the tropological fabric of history, he simultaneously questioned two constitutive moments of traditional historiography: its impartiality (Ranke’s famous *Wie es eigentlich gewesen ist*) and its dissociation from literature. However, to better understand the far-reaching nature of White’s insights, it is useful to compare him with another (no less controversial) theorist from a different field. White’s position is not as idiosyncratic as it seemed to some of his colleagues, and the striking similarity that can be seen between his tropological perspective and the one advocated by the political theory of Ernesto Laclau will allow us, in the next chapter, to place White’s investigations in a complementary relationship with those of post-foundational political theorists to emphasize the higher degree of generality of his insights. Moreover, in the chapters that follow the second chapter, we will explore whether the contributions from the rich corpus of research on the politics of literature that have emerged on the basis of post-foundational theory can complement the conclusions about the epistemological and political implications of sensitization to the literariness of history that White and other theorists who share his perspective put forward. In other words, orbiting around the notions of tropology and literariness, we will first examine the important correspondences between White's theoretical claims and post-foundational political theorists, and then ask how the different approaches to the politics of literature can contribute to thinking the literariness of history.

## II.

White’s insistence on the fundamentally tropological nature of history, temporarily grounded and thus obscured by the prevailing ethos of a particular community ^3^, can easily be recognized as a variant of what Oliver Marchart ( 7) calls “post-foundational political thought”. In contrast to foundationalism, which believes in the existence of a present and final foundation of society, or anti-foundationalism, which negates any foundation, post-foundational thought is characterized by an ontology that rests on a foundation that is simultaneously absent and necessary/presupposed ( 7 on page 164), leading to the necessity of political, contingent, and essentially tropological grounding ^4^. According to Marchart, Laclau’s political theory is the epitome of such paradigm, since “(a) Laclauian hegemony or discourse theory is, essentially, a political ontology; and (b) such political ontology must assume the status of a prima philosophia or ‘first philosophy’ (in a qualified sense, of course).” ( 7 on page 146)

Such an equation already existed in Laclau’s earlier works. In *Hegemony and Socialist Strategy* ( 8), he introduced the discursive conceptualization of the socio-political field and referred to the operations in this field with rhetorical terms. Instead of a firmly grounded sutured society with fixed social actors, he outlined the semi-permeable field of the social, permeated by various antagonisms, in which socio-political actors are formed along the lines of these antagonisms through contingent hegemonic articulations. Laclau, who, like White, was a reader of Roman Jakobson, described these articulations using two basic tropes: metonymy (for the initial hegemonic relation in which a sociopolitical actor steps out of its differential position in society and subsumes other actors under its umbrella) and metaphor (for a telos of hegemonic articulation, a chain of analogy organized along a particular line of antagonism).

In his last book, *The Rhetorical Foundations of Society* ( 10), Laclau went even further by entirely reformulating his political theory in rhetorical terms. The conviction that there is no zero point of figurality, that is, no underlying objective truth of social relations obscured by ideological representations, that socio-political articulations are structured as metonymies and metaphors, and that every act of grounding a social system is a catachrestic investment, leads Laclau in this book to claim that politics is about “articulation of heterogeneous elements, and such an articulation is essentially tropological.” ( 10 on page 67) To describe the fundamental equivalence that precedes any articulation, Laclau draws on the famous formalist concept of literariness. He argues that “conceived at such a broad level of generality, the literariness (…) breaks the limits of any specialized discipline, and its analysis involves something like the study of the distorting effects that representation exercises over any reference – effects that thus become constitutive of any experience.” ( 10 on page 79)

Although White and Laclau undoubtedly differ in terms of the emphases of their works, their disciplinary contexts, and their political positions, analogous assertions regarding the tropological articulation of what is presented as objective historical reality are significant and owe much to similar theoretical impulses in their intellectual backgrounds. Moreover, in both cases, the catachrestical grounding of such representation is political *par excellence* and closely linked to literariness conceived at a very general level. Laclau, however, is not the only political theorist who draws on such a general concept of literariness.

Jacques Rancière, whose political theory, despite some discrepancies, is highly compatible with Laclau’s, also argues that “the modern political animal is first a literary animal, caught in the circuit of a literariness that undoes the relationships between the order of words and the order of bodies that determine the place of each.” ( 11 on page 37) Therefore, according to him, “the democratic man is a being who speaks, which is also to say a poetic being, a being capable of embracing a distance between words and things which is not deception, not trickery, but humanity; a being capable of embracing the unreality of representation.” (Rancière 12 on page 51)

In contrast to Laclau, Rancière also dealt with history, most famously in his study *The Names of History* ( 13), whose foreword was written by none other than Hayden White. The uncoupling of words and things, which is recognized in his political theory as a prerequisite for the (modern) political being, is also invoked here as a necessary condition for the existence of history. “There is history”, writes Rancière, “precisely because no primeval legislator put words in harmony with things.” ( 13 on page 35) He expresses this argument in an explicit polemic against various “scientific” tendencies in historiography, whose common denominator is the attempt to eliminate this “anachronistic and homonymic confusion” ( 13 on page 34) and to overcome (or at least reduce) the notoriously unscientific narrative component of history.

This “anachronistic and homonymic confusion” necessarily arises from the anachronistic nature of historical event ( 13 on page 30), and “requires that the words of history be names” ( 13 on page 34) rather than classes in the scientific sense (which are strictly determined by a set of essential properties), which is just another way of arguing that (historical/social) actors emerge through tropological articulation, not through the recognition of certain objective shared characteristics, and that the narratives which provide space for their articulation are necessarily products of allegoresis ^5^. Rancière, who is even more intensely concerned than White with questioning the epistemological status or “poetics of knowledge” of narrative history, is firmly convinced that it is because (and not in spite of) “[it] has strictly maintained itself in the space of homonymy of science and science, because it has kept the name of the stories told to children and of the community legends taught to students in school, history has been able to lead straight to the impossible task of articulating three contracts in a single discourse” ( 13 on page 9), namely scientific, narrative and political.

The absence of a “primeval legislator” – the absent yet necessary ground that characterizes post-foundational thought and makes such a tripartite articulation possible – and the resulting proliferation of excess, which Rancière metonymically embodies in his work *The Names of History* in various situations such as the death of the king, the deposition of the established order, or the revolutionary event ( 13 on pages 21, 30, 37 etc.), resonates in this study with emancipatory, progressive connotations. This fits well with the general situation within post-foundational thought, but White’s perspective on the same problem is not clear-cut. On the one hand, White repeatedly praises the ironic approach of abandoning blind faith in various grand narratives and jettisons the notion of predetermined historical goals and meanings; he even explicitly states that his endeavor is ironic in this sense. On the other hand, he argues that the predominance of the ironic approach contributed significantly to the crisis of historical consciousness in the 19th century, from which professional historiography still has not recovered ( 3 on page xii).

This apparent contradiction appears less contradictory if one takes into account, as Paul ( 9 on page 37) notes that in White's *Metahistory* ( 3), there are at least four different meanings of irony, not as a result of inconsistency, but of the complexity of the problem. In his view, the contradiction can be resolved primarily by distinguishing between epistemological irony (which White uses and praises) and ideological irony (as criticized in the writings of Burckhardt and Croce, for example) ^6^. However, while Paul’s observation is generally correct, the distinction between the two is difficult to make in practice. It is often difficult to draw a clear line between epistemological skepticism and ethical/political relativism.

The problem is not limited to history; it is only a specific variant of a more general issue concerning post-foundational theory, which is regularly criticized from the perspective of various foundational theories for its inability to avoid succumbing to relativism. Referring to Derrida’s reading of Husserl, Laclau countered this criticism by defining hegemony as “a theory of the decision taken in an undecidable terrain”, which “requires that the contingent character of the connections existing in that terrain is fully shown by deconstruction” ( 15 on page 90). This awareness of the necessity of engagement, catachristic investment, and decision taking in an undecidable terrain is then – as already mentioned – what distinguishes the post-foundational from the anti-foundational position. It is true, however, that many post-foundational theorists – including occasionally Laclau and Rancière, and arguably White – emphasize above all the de- versus the con- component of deconstruction, that is, they focus largely on what is perceived as the emancipatory moment of undoing a particular structure and exposing its contingent character, forgetting that this is necessarily followed by the establishment of another structure (no matter how contingent and catachrestic it is).

The necessity of the latter is perhaps brushed upon by White in *Metahistory* ( 3) in his desire for the resurgence of metaphorical understanding of reality (cf. Paul 9 on page 36). However, this desire may seem like a nostalgic lament for a lost paradise and is clearly overshadowed in White’s later works by focusing on the study of modernist representational techniques. ^7^ However, similar concerns regarding the saturation of ironic consciousness have arisen in various fields, including literary criticism. Contemporary discussions of the politics of literature resonate with analogous themes and revolve around the questions that White raises within the historical field, especially in his later works. Since the points of contact between literature and history obviously go beyond the mere formal kinship of the two and the common archive of modes of emplotment they employ and revolve around the generalized notion of literariness, which is also crucial to post-foundational political thought, it would be useful to focus on these discussions and confront them with White’s dilemmas concerning the representational strategies of historical accounts.

## III.

The concept of the politics of literature is notoriously elusive because it suffers from a homonymy similar to that of history. For a long time, this topic has mainly been viewed through the prism of the engaged literature. However, Marxist ideological criticism on the one hand and the avant-garde/formalist focus on literary estrangement on the other have added additional angles that are not necessarily mutually exclusive. Interestingly, one of the most concise overviews of the various forms of engagement between literature and society, that is, of the various ways of conceptualizing the politics of literature, comes not from a literary theorist but from the historian Dominic LaCapra.

In his work *History, Politics and the Novel* ( 16 on page 1), LaCapra states that he does not adhere to the “theoretical principle or aesthetic conviction that in literature only the indirect relation to politics is legitimate”, i.e. he concedes that “the direct, event the militantly didactic [approach] (…) may have its place at least insofar as it does not descend to the level of manipulative propaganda.” However, this blatant embodiment of the politics of literature is by no means the only possible one. Indeed, he warns that “the great temptation in recent ‘political’ readings has been to interpret all cultural artifacts predominantly if not exclusively, as symptomatic expressions of dominant discourses and historical pressures” ( 16 on page 2), and he opts for a more complex and analytically subtle perspective on this problem. For this reason, he rejects both vulgar contextualism and radical immanentism, and claims that the relationship between literature and history can take roughly three forms: symptomatic, critical, and transformative. The first form is a more or less sophisticated version of reflection, in which literature is subordinated to history and merely reflects or reproduces established tropological relations. On the other hand, in the second two cases, the literary text is granted more agency as it either reveals or even partially undermines latent ideological mechanisms.

These relationships are not mutually exclusive; they often coexist in the novels he analyzes. However, LaCapra’s preference for more sophisticated modernist narrative strategies is evident both in his analyses (in which he deals primarily with modernist literary works) and in explicit assertions such as that the text “may have transformative effects more through its style or mode of narration than in the concrete image or representation of any desirable alternative society or polity.” ( 16 on page 4) Despite their occasional divergences, LaCapra is here on the same page as White, who in his later works also explores the importance of narrative devices and strategies and draws on modernist literature for examples. In his essay on Droysen, for example, White practically mirrors LaCapra’s stance mentioned above, claiming that “the ideological element in art, literature, or historiography consists of the projection of the kind of subjectivity that its viewers or readers must take on in order to experience it as art, as literature, or as historiography”. On the one hand, “art and literature have a domesticating effect when they project as possible subjectivities for their consumers the figure of the ‘law-abiding’ citizen” ( 2 on page 87), while on the other hand they

become “revolutionary” or at least socially threatening, not when they set forth specific doctrines of revolt or depict sympathetically revolutionary subjects, but precisely when they project – as Flaubert did in Madame Bovary – a reading subject alienated from the social system of which the prospective reader is a member. (White, 2 on page 87)

White thus asks, in general, whether it is possible to imagine a conception of history that leaves behind the realist narrative conventions and their ideological implications, that would “signal its resistance to the bourgeois ideology of realism”, and whether such a history enables "a recovery of the historical sublime that bourgeois historiography repressed in the process of its disciplinization?” ( 2 on page 81) This question becomes even more pertinent when it comes to what he calls “modernist” or “holocaustal events” that emerge in the course of the 20th century and which, because of their scale and “anomalous nature”, cannot be adequately captured and represented in the categories inherited from realist narrative modes ( 4 on page 70). However, is it really possible for history to abandon such realist modes of representation and remain recognizable as historical discourse? What models do the (modernist) literature offer viable alternatives?

Returning to LaCapra’s remarks about the politics of literature, it is relatively easy to see the counterpart to the rather blatant politicalness of overtly engaged literature in equally overtly engaged historiography, such as various counter-histories (e.g., Zinn’s *People’s History of United States* ( 17) or some forms of history from below, with no intention of diminishing the value and professional standards of such works. To paraphrase White: What was recognized as tragedy elsewhere might be recognized as farce here; what was recognized as freedom elsewhere might be recognized as tyranny here, etc. However, what about the “transformative effects” based on different formal means rather than different images ( 16 on page 4)? Is it conceivable to represent something *simultaneously* as tragedy and farce or as freedom and tyranny? Is it possible – as LaCapra and White often do – to find an answer to this question in the literary field?

If we remember that, according to White, historical narrative is primarily about interpretation and not explanation, i.e. that “/t/ragic, comic, epic and farcical are not categories descriptive of real events”, but are “at best interpretive”, and that certain events can be “tragic or comic or epic or farcical only when viewed from the perspective of the interests of specific agents or groups involved in them” ( 6 on page 487), and not because of their true nature, a detour to Soviet literary criticism of the 1920s might be useful for further discussion. In his work *Discourse in Life and Discourse in Art*, Valentin Voloshinov ( 18) argues that every utterance contains a third element in addition to form and content: an evaluation, a component that is social par excellence, a kind of ideological judgment in the broader sense that organizes both form and content. The evaluations which “have entered the flesh and blood of all representatives of the group” ( 18 on page 166) usually remain imperceptible, completely fused with the object of the discourse, which corresponds to the imaginary zero point of figuration in the tropological perspective on the discourse. Notable exceptions are times of social turbulence, when implicit evaluations suddenly no longer feel natural, and the ideological determination of each discourse becomes visible again. However, since literary texts are necessarily detached from the immediate communicative situation, Voloshinov believes that “a poetic work is a powerful condenser of unarticulated social evaluations – each word is saturated with them. It is these social evaluations that organize form as their direct expression.” ( 18 on page 178)

This is particularly noticeable in the case of the polyphonic novel, as described by Voloshinov’s close collaborator Bakhtin ( 19). As the product of a historical moment of instability in which “social multiaccentuality” is dramatized on different levels and by different literary devices ^8^, the polyphonic novel can be seen as a special case within a special case – a literary phenomenon that is particularly effective in revealing the tropological fabric of society and potentially reconfiguring its elements. At the same time, it is the literary phenomenon that comes closest to the common denominator of several attempts to define the elusive genre of the political novel since Howe’s study *Politics and the Novel* ( 20). Howe argues that

like a nimble dialectician, the political novelist must be able to handle several ideas at once, to see them in their hostile yet interdependent relations and to grasp the way in which ideas in the novel are transformed in something other than the ideas of the political program. ( 20 on page 23)

The ideas that the novel “appropriates are melted into its movement and fused with the emotions of its characters”, which “stir characters into passionate gestures and sacrifices” ( 20 on page 23). Given the influence Bakhtin’s conceptions had – directly or indirectly – on Howe, it is no wonder that he singles out Dostoyevsky’s *The Possessed* as “the greatest of all political novels” ( 20 on page 24). Similarly, if we return to LaCapra’s analysis of the various ways in which the novel can have a political effect, it is not surprising that he admits:

/i/f there is one general notion if not ‘theory’ of the novel that is especially active in my analyses, it is Mikhail Bakhtin’s understanding of the novel as a self-contestatory, carnivalizing genre that tests the limits of generic classification and enacts a dialogical interplay of often dissonant ‘voices’ and ideological currents. ( 16 on page 208)

Nevertheless, the question remains, in view of the kinship between the novel and narrative history, or despite this relationship, whether novelistic polyphony as conceived by Bakhtin is possible in historiographical discourse ^9^.

Pihlainen ( 5) and Morson ( 21) took up this question and tried to answer it positively by proposing a rather peculiar option. In contrast to the traditional historical narrative, Morson argues that a

better alternative, one much more consonant with the thinking of Tolstoy, Dostoevsky, and Bakhtin would be to imagine the what-ifs—several of them, most likely—and then follow the choice that was actually made; and at the next moment of choice, do the same, repeatedly. In that case, one would have a sense of history as constantly presenting alternatives and the history we know as one possibility among legions. ( 21 on page 69)

It is difficult to see, however, why a repetitive recourse to what-if thinking should go hand-in-hand with Bakhtin’s notion of polyphony. This strange prescription could be the result of a complete misunderstanding of what Bakhtin’s polyphony implies, for Morson also claims:

Bakhtin described the polyphonic novel, which I regard as one type of literature of process, as a work in which the author achieves eventness by surrendering the “essential surplus of meaning” provided by knowledge of the work’s structure and placing himself on a level of ignorance with the characters. Characters do not choose and act in execution of a plan of which they know nothing, like John Milton’s Satan, because the author knows what they will do only when they do it. The events take place “right now, in the real present of the creative process”. ( 21 on page 72)

Bakhtin’s assertion that the character in the polyphonic novel is “not an object of authorial discourse, but rather a fully valid, autonomous carrier of his own individual word” ( 19 on page 5) has nothing to do with “events taking place right now, in the real present of the creative process” ( 21 on page 72), but with the contrapuntal nature of each level of the novel; the heterogeneity of contrasting materials and styles, narrative perspectives and voices, characters and their ideas, and so on.

Since the realization of novelistic polyphony understood in this way – in addition to the heterogeneity of the included material – largely depends on the (variable) internal focalization ( 23 on page 189), Genette’s observation of the rarity of typical internal focalization in what he calls factual narratives ( 24) casts doubt on the possibility of its employment in historiographical discourse. This doubt is shared by David Perkins, who, although writing about literary history, argues that the bias of authorial perspective cannot be avoided in historiography as it can in novelistic discourse, since “modernist forms of narration have not been exploited in literary history and cannot be adapted to its purposes.” ( 25 on page 31) The only way he sees as viable to preserve the plurality of perspectives and styles is the so-called encyclopedic form ( 25 on page 53), which brings together texts by various authors with various approaches and styles on a common theme. However, although this form retains certain narrative qualities, its narrativeness is diminished, making it an alternative rather than a variant of narrative history.

In contrast to Perkins, however, most of the scholars mentioned (Rancière, White, LaCapra) went a step further than the Dostoyevskian polyphonic novel in their inclination towards modernist literary devices in their search for solutions to the pitfalls of traditional historical narration. In doing so, they moved close to another influential but controversial perspective on the politics of literature.

## IV.

One of the most recent theoretical embodiments of LaCapra’s third type of relationship between literature and history – the one in which literature intervenes in a less blatant way in the fabric of extra-literary reality, namely through specific literary devices – is certainly Rancière’s famous conception of the politics of literature. According to him, the politics of literature is “not the politics of writers” nor is it the mere “representation of social structures, political movements or various identities in literary works” ( 26 on page 11) Rather, by “politics of literature” we should understand a literature that engages with politics “by remaining literature”; that is partaking in the division of the sensible, making visible what was invisible, giving a voice to those who had none, etc. ( 26 on page 12)

This perspective derives from Rancière’s general definition of politics, most famously expressed in *Disagreement* ( 11). In contrast to conventional usage, in this study he uses the term “police“ to refer to the procedures that organize power and distribute places and roles in a given system ( 11 on page 28), while reserving “politics” for any activity that “shifts a body from the place assigned to it or changes a place’s destination. It makes visible what had no business being seen, and makes heard a discourse where once there was only place for noise...” ( 11 on page 30).

Described in this way, the reconfiguration of the order of sensible in the literary field can take a wide variety of forms. In fact, when explaining the so-called radical democracy of writing, the equality of themes and styles ( 26 on page 21) that breaks with the old regime of *belles lettres*, and that is nothing other than literariness generalized in the same way as in Laclau, Rancière describes the interplay of three (conflicted) levels, ranging from the inclusion of previously excluded social classes with their specific styles and themes in the literary field to the focus on the “micro-events or individualities that no longer constitute individuals but differences in intensity, whose pure rhythm cures every social fever.” ( 26 on page 35) In his later works, however, his analytical preference shifts noticeably to the latter category, to which he devotes a considerable part of his investigations. This was the target of harsh criticism, in which several scholars objected to his preference for modernist devices and the alleged inflationary backlash of his overly heroic vision of literature, according to which almost everything in literature can be considered political and emancipatory (and consequently nothing specifically is).

Without going into the details of these objections, another problem with Rancière’s preference for “molecular” over “molar” ( 26 on pages 35, 50) phenomena from a historiographical perspective is that it diverts attention from the (individual or collective) actors and their actions emplotted in teleogenetic plots, central to traditional historical narratives (and whose perspectives, according to White ( 6 on page 487), are essential to the tropological articulation of historical meaning), and focuses more on the fragmented and undifferentiated intensities typical of the modernist narration. It seems challenging, then, to imagine how to deploy this kind of democracy of writing in historiography.

In his later works, however, in which he discusses the specific nature of “modernist” events that elude the representational capacity of the traditional historical narration, White writes affirmatively about modernist narrative strategies as possible solutions to this problem in a way that is very reminiscent of Rancière. He argues that

modernist literary practice effectively explodes the notion of those characters who had formerly served as the subjects of stories or at least as representatives of possible perspectives on the events of the story; and it resists the temptation to emplot events and the actions of the characters so as to produce the meaning-effect derived by demonstrating how one’s end may be contained in one’s beginning. ( 4 on page 74)

That is why, according to White, literary modernism can be seen as “a product of an effort to represent a historical reality for which the older, classical realist modes of representation were inadequate, based as they were on different experiences of history or, rather, on experiences of a different history.” ( 4 on page 41) ^10^ He also emphasizes that the strategies of modernism are a further development of realist narration rather than its radical negation ( 2 on page 168; 4 on page 99).

Nevertheless, White’s affirmation of modernist narrative strategies as a response to the inadequacies of traditional monological historical narration in the face of modern social and political challenges remains at a rather general level, without many examples from concrete historiographical practice, more as a task for future historiography than as something that has already been successfully employed. In contrast, Rancière believes that such strategies had already been successfully applied at the very beginning of modern historiography.

Rancière’s initial stance mirrors White’s quite closely, for he too claims that

to get out of the desperate dilemma between the illusion of the popular *epos* and the rigors of number or the minutiae of the everyday, one had to attach oneself to the new logic invented by literature to hold together the paths of the individual and the law of number, the little glimmers of the everyday and the flame of the sacred text. ( 13 on page 100)

The inspiring literary examples that history should draw on are modernist authors such as Woolf, Flaubert or Joyce. However, according to Rancière, the first step in the process was already taken in an earlier epoch. The heroic figure in Rancière’s *The Names of History* is none other than Jules Michelet, the French historian to whom White also dedicated the first analytical chapter of his *Metahistory*. However, Rancière’s perspective on Michelet is somewhat different from White’s. Rather than focusing, as White does, on the features of his writing that helped to establish the realist, mimetic canon of nineteenth-century historiography, Rancière treats him as a proto-modernist, narratively innovative writer. He argues that “Michelet invented a poetics for a certain historicity” ( 13 on page 101), and in this poetics he recognizes all the crucial topoi of what he will later call radical democracy of writing.

This is why Rancière claims that the success of Michelet’s history does not occur “in spite of the excess of romanticism; it wins in the very heart of the movement called romanticism, which first of all signifies the end of the mimetic reign and the transformation of the rules of belles lettres into the unconditioned of literature.” ( 13 on page 51) The new artistic regime established by literary modernity allowed Michelet to make room in history for the presence of the previously excluded, but not by following the rules of appropriateness and adapting their voices to the standards of the previous discursive regimes, as the classical historians already did with the voices of the slaves or the plebs: “He invents the art of making the poor speak by keeping them silent, of making them speak as silent people.” ( 13 on page 45)

The arsenal of devices for such a Copernican turn is rich and entirely owed to the “radical democracy of writing”, that is, pure literariness, the equality of themes and styles that modern literature has introduced. It bypasses “the public writers, the village scholars, or the pedants who assume the task of composing the letters of the illiterate” ( 13 on page 46) and ranges from the introduction of hitherto invisible themes (e.g. the history of popular heresies rather than organized belief systems), the focus on the meaning of the document as an event rather than its content, the exchange of narratives about the events for narratives about the meaning of the events, the use of vivid metaphors that activate all the senses and thus make things perceptible, to complex tropological constructions that give voice to estranging, metaphorically formed agents (often even inanimate or abstract ones), such as mud, roads, sidewalks, walls, air, fire, festivals, generations, etc.

The paragraphs from Michelet such as:

No, do not believe it. Nothing is forgotten – neither man, nor thing. What once has been, cannot be thus annihilated. The very walls do not forget, the pavement will become accomplice, and convey signs and noises; the air will not forget.

or

This prophet and holy fool is not a man like other men. What speaks is a whole city, a whole bleeding world – the agonized cry of Lyon. He is the voice of the deep dark mud of its streets, silent since the beginning of the time. Through him the ancient, dismal darkness, the damp and filthy houses begin to speak; and hinger and fasts; and abandoned children and the women dishonored; and all those heaped-up, sacrificed generations.

cited by White White ( 3 on page 156) and Rancière ( 13 on page 47) are structurally analogous to the epitomic examples from modernist novels that Rancière cited in several of his studies, including *Politics of Literature* ( 26). Moreover, he even follows Michelet’s style as a blueprint in his own attempt to give voice to the voiceless, writing his historical study *Proletarian Nights* ( 14) in an unmistakably modernist manner.

However, there are some obstacles that prevent us from agreeing with Rancière’s proposal to take Michelet’s writing unreservedly as a model for history that recognizes and uses its own tropological nature while allowing a multiplicity of voices to speak. To begin with, the “excessive” feature of Michelet’s discourse, which Rancière clearly sees as the core of the solution, dances on the edge of what the professional public can recognize as valid historiography. This is reflected in the status assigned to Michelet today, whose importance as a historian is widely recognized but whose style is overwhelmingly regarded as overly romantic and unsuitable for contemporary historiography. The same applies to Rancière’s *Proletarian Nights* ( 14), which has never been widely recognized in professional historiography.

But even if we leave aside the question of professional trends and assume that thorough rearticulation of historiographical discourse is possible, a theoretical problem remains. Rancière argues that a logical characteristic of the politics of literature is the logic of misunderstanding ( *la malentendu*), while the logic of politics proper is the logic of disagreement *(la mesentente*). Although both logics have the important commonality of disrupting the established order that binds words and things together, their modus operandi differ. While disagreement establishes new collective/subject positions, misunderstanding functions ”by suspending the forms of individuality by which consensual logic ties bodies to meanings” ( 26 on page 52). In other words, according to Rancière, the specific politics of literature–that is, misunderstanding as its primary logic–operates primarily by undermining and dismantling tropological systems without seeking to establish another, as opposed to disagreement, in which the previously excluded subject challenges the actual order in order to reconfigure it and establish its own position.

This exclusively “molecular”, transgressive, emancipatory nature of misunderstanding is the main target of the critiques of Rancière mentioned above. However, without addressing the question of their validity, one thing is certain: it is doubtful that this logic can be applied to Michelet. First, Rancière himself praises Michelet in *The Names of the History* ( 13) for giving voice to previously excluded historical actors, which would place his writing in the realm of disagreement rather than misunderstanding. Second, White’s analysis of Michelet shows that his discourse does not cancel out all tropological articulations but rather affirms a particular one. In some ways, White is also particularly fond of Michelets. According to Paul ( 9 on page 36), his lament for the crisis of nineteenth-century historiographical consciousness caused by the dominance of irony is complemented in *Metahistory* ( 3) by the conviction “that historians should fight this irony with its own weapons in order to crate an opportunity to ‘return’ to a metaphorical comprehension of reality.” The epitome of 19th century metaphorical understanding is none other than Michelet.

Although claims such as the one that

Michelet dissolved all sense of difference among men, institutions, and values. His Metaphorical identification of thins that appear to be different utterly overrode any sense of the difference among things, which is the occasion for Metaphorical usage to begin with. All difference was dissolved in his apprehension of the unity of the whole. ( 3 on page 157)

may sound like a confirmation of the Rancièrian democracy of writing, that is, generalized literariness or absolute equality of language, the situation is different. White explicitly situates Michelet within certain historical coordinates, associates him with certain political ideologies (somewhere between liberalism and anarchism ( 3 on page 162)), and shows that at the end of Michelet's gradational chain of metaphors is a concrete political entity – the French Republic. In other words, and in Laclauian terms, although Michelet succeeds in creating impressive chains of equivalences that seem to abolish all boundaries and inequalities, there is a transcendental signifier that fixates this chain. And although one could argue that France here is as an empty signifier for radical democracy as justice, fraternity, unity, or equality, which Michelet also invokes ( 3 on page 151), this empty signifier is charged with concrete historical content. Here, we deal with the textbook case of Laclauian catachrestical investment that grounds the tropological system; seemingly universal signifiers are hegemonically embodied in incommensurable singularities.

However, if, contrary to the hopes of White or Rancière, the use of modernist narrative strategies does not necessarily lead to a subversion of a certain established discursive order, and if it does not of itself prevent history from becoming an instrument for its reproduction and the production of compliant subjects – what other possibility does history have of making itself more political in the Rancièrean sense?

In historical studies, a number of attempts have been made to answer this question with the help of various methodological concepts, such as microhistory or the history of everyday life ( *Alltagsgeschichte*). However, one of the most innovative and comprehensive attempts was made by a historian whose work emerged at the intersection of more traditional history, its poststructuralist critique, and various other disciplinary influences (of which psychoanalysis was perhaps the most influential), Michel de Certeau. Without going into a detailed account of De Certeau’s positions, it is important to note that he shares with authors such as White and Rancière a critical awareness of the tropological nature of history, a consciousness of its entanglement with power relations, and a desire to make it more pluralistic and emancipatory. Nevertheless, it is perhaps not wrong to say that he has a somewhat keener ear for the institutional context in which history functions.

In his *Writing of History*, De Certeau repeatedly points to the close relationship between history and power, emphasizing how the situatedness of history in geographical and discursive spaces determines the choice of themes and perspectives. Furthermore, he argues, similar to White, that history can be “envisaged as a text organizing units of meaning and subjecting them to transformations whose rules can be determined.” ( 27 on page 41) ^11^ However, while on the one hand appreciating the Foucauldian perspective on the connection between discourse and the microphysics of power, De Certeau was on the other hand interested in recognizing and examining the equally capillary vectors of resistance.

The most famous example of such an endeavor is De Certeau’s conceptual opposition between strategies and tactics. The former are institutionalized practices with elaborated theoretical backgrounds, procedures, social positions, and so on; the latter refers to numerous non-institutionalized, ad hoc, statistically and systematically invisible practices that circumvent or bend the established framework in their everyday enactments, that is, re-semantize the prevailing tropes. Alternatively, to put it more technically,

Strategies are actions which, thanks to the establishment of a place of power (the property of a proper), elaborate theoretical places (systems and totalizing discourses) capable of articulating an ensemble of physical places in which forces are distributed. They combine these three types of places and seek to master each by means of the others. (…) Tactics are procedures that gain validity in relation to the pertinence they lend to time-to the circumstances which the precise instant of an intervention transforms into a favorable situation, to the rapidity of the movements that change the organization of a space, to the relations among successive moments in an action, to the possible intersections of durations and heterogeneous rhythms, etc. ( 28 on page 38)

Shifting the historian’s interest from strategies to tactics, or more precisely to their interplay, could be another way of writing a history that examines and subverts the dominant framework, much as a political novel examines and subverts it in the literary field, while keeping in mind the difference between the two institutions. However, as De Certeau knows, this implies a shift not only at the thematic level, as the mapping and investigation of tactics requires a specific research optic and discursive means that are themselves more tactical in nature.

Moreover, there is something in this transition from strategies to tactics that is yet again connected to the Rancièrian radical democracy of writing–that is, with generalized literariness. De Certeau ( 28 on page 70) points out that in a time of intense technologization and rationalization, a kind of “remaining ways of operating” have survived that have “no legitimacy with respect to productivist rationality”. These elements, condemned to “the margins or in the interstices of scientific or cultural orthopraxis”, found their way into literature, beginning with the realist novels of the 19th century. “Literature is”, thus, “ transformed into a repertory of these practices that have no technological copyright. They soon occupy a privileged place in the stories that patients tell in the wards of psychiatric institutions or in psychoanalysts' offices.” ( 28 on page 70) In other words, narratives that circulated on the margins and outside the normalized space, and thus could violate the rules of appropriateness, became privileged “containers” for the “narrativity for everyday practices”; archives and nodes of trajectories that escape the framework of strategies and their seats of power.

De Certeau also recognizes that this equality in language, a literariness that uncouples words and things ( 12 on page 51), subverts the established elites and their social power by exposing the illusoriness of “literal meaning”, that is, by revealing that all meaning is a hegemonically established tropological articulation. Observing things from the opposite perspective and focusing on the guardians of the imposed order, he writes:

The use made of the book by privileged readers constitutes it as a secret of which they are the "true" interpreters. It interposes a frontier between the text and its readers that can be crossed only if one has a passport delivered by these official interpreters, who transform their own reading (which is also a legitimate one) into an orthodox "literality" that makes other (equally legitimate) readings either heretical (not ‘in conformity’ with the meaning of the text) or insignificant (to be forgotten). From this point of view, "literal" meaning is the index and the result of a social power, that of an elite. By its very nature available to a plural reading, the text becomes a cultural weapon, a private hunting reserve, the pretext for a law that legitimizes as "literal" the interpretation given by socially authorized professionals and intellectuals (clercs). ( 28 on page 171)

Despite all the differences in their approaches and various shortcomings that become visible when they are placed in relation to one another, the recognition of history as a tropological articulation and literariness as that which enables and subverts it thus remains an indispensable element in the attempts of all the authors analyzed to rethink the specific logic of politics of history. Consequently, it seems that maintaining a connection with the developments of literature and its devices is a reasonable and perhaps even inevitable way to make history more critical and pluralistic, but by no means a magic wand. However, is literature really a heroic emancipatory discourse *per se*? How far can this tendency go without undermining the particular formal characteristics and social roles of both discourses?

## V.

Rancière ( 13 on pages 7, 9) insists that a specific interplay of homonyms of history as science and as narrative and its positioning in the ambivalent space between science and non-science and close to literature is what keeps history not only self-critical, but also alive and independent from the efforts of the social sciences to absorb it. However, contrary to his intentions, interpretations and elaborations of this argument sometimes give the impression of an overly heroic and asymmetrical role of literature in this relationship, as an inexhaustible source of necessary devices and as an avant-garde sister that history can (and should) always look up to. From this perspective, certain things can be easily forgotten. First, when reading Rancière's, White's, or LaCapra's appraisals of the estranging and effective modernist literary strategies, it is easy to forget that no literary device has certain effects – or is even recognizable–on its own. It is the background of the conventional on which we experience estranging, and the background of the extra-literary context on which we experience literature. If the historiographical discourse is the one to which it is due that we understand the extra-literary, the stabilized “literal” meaning, the “conventional(ized)” techniques of representation, a provisional zero point of figuration, which the (differently understood) politics of literature exposes as contingent – then the effect of all supposedly politically potent literary devices and strategies depends on their declension from the conventional, and there is only a certain scope for bringing history and literature closer together without uncritically ignoring and consequently imploding the difference between the two institutions.

This insight has been reflected in the form of appeals by various scholars against overly enthusiastic literalization of history under the influence of pioneering literary theoretical insights and the appeal of certain (especially confessional) literary forms, which disregard the institutional specificities of the two discourses and one-sidedly and somewhat naively privilege the literary one. As Vladimir Biti ( 29 on pages 20, 26, 45) notes, this ranges from De Certeau's and LaCapra's observations on the multiple contextual factors in determining historical discourse, in which they drew on French theory, to the work of German neo-Enlightenment theorists of history.

Second, what Biti ( 29) also warns that this uncritical glorification of the literary in history has certain paradoxical effects. The function of the modernist narrative strategies and devices praised by White, Rancière or LaCapra was to heighten the critical self-reflexivity of history, that is, to expose the tropological system of mediation at the point where immediate access to objective, literal meaning was conventionally seen. However, in the later heroic treatment of the literary component in history, literature was privileged primarily because it was seen as a less-standardized discourse that was closer to unadulterated reality. In other words, instead of exposing the hegemonic tropological structures and general literariness in their background that constantly subverts them, the focus was shifted to normalizing a particular tropological configuration and equating literariness with it. The epistemological irony that is fundamental to White’s endeavor is thus not hypertrophied but discarded altogether.

 Although this development is often attributed to an over-theorization of history, or at least to an over-inclusion of literary theory in this theorization, it is important to note that (reasonable) appeals against it are not appeals for less, but for more theory. A good illustration is provided by the notion of experience, one of the epitomes of the highly prized alleged immediacy and non-standardization. LaCapra objects to the inflationary use of this concept and is even wary of microhistory and history from below, when they introduce certain concepts and conclusions too casually:

At times "experience" threatens to become a hollow shibboleth, especially when what begins as populism turns into an indiscriminate methodology, and one affirms the need to recover lost popular voices in cases marked by insufficient evidence of any sort and the tendency to compensate for such insufficiency through unrestrained speculation, projective identification, and ventriloquism. In any event, "experience" is a frequently invoked but undertheorized concept both in history and in related disciplines or discourses, and much remains to be done in its critical examination and use and in elucidating its relation to structural as well as institutional analyses of society, culture, and the complex vicissitudes of trauma. ( 30 on page 4)

Similar concerns were set out in more detail by Nenad Ivić in his study *Civil War of Words* ( 31). In discussing the relationship between literature and history, Ivić's study firmly opposes the ideas about their hybridization raised by Jablonka ( 22, 32) but also examines the institutional vectors that facilitate such tendencies. He argues that the driving force behind such visions is not epistemological but rather market logic. Ivić writes, not without a subtle irony, that “in a world in which, as it is believed, literature of experience reaches wider audiences, or better sales, than the experience of literature, the historian wants to break out of the narrow confines of his professional, institutional, and thematic market prohibitions and enter the global marketplace of words.” ( 31 on page 51)

This desire urges historians to render history vivid, close, and accessible ( 31 on page 51). However, as Ivić notes, this has a paradoxical effect: since success depends on “arousing everyone's emotions”, it comes at the cost of “depriving history of its interestingness, which lies in the particularities, details and differences”. In this way, ”success, which should enable the historian to be heard, robs his work, his bestseller, of what should be heard: History as knowledge.”( 31 on page 53) In other words, “knowledge is structured as martyrology” ( 31 on page 54); belonging (real or imagined) to one of the disadvantaged groups and bearing direct witness to this experience becomes synonymous with scholarly legitimacy, and the skepticism that seeks to warn against oversimplification becomes politically and professionally suspect. Paradoxically, this is an analogous situation to the one in which skepticism was equally suspect when it aimed to emphasize the literariness of history and opted for a more literary approach. What is scandalizing in both cases is a certain critical awareness of the nature of the historical discourse, of the presuppositions of its meaning, its immanent characteristics, and its broader institutional context. An awareness that, if lost, easily slips into the advocacy of this or that unreflective approach.

If White has taught us anything, it is that (epistemological) irony is crucial to avoid simplifications that overlook the tropological fabric of history, regardless of their differently oriented embodiment. At the same time, all this confirms Ivić's assertion that the reality effect of history “cannot be achieved without the invention of language (…), the invention that brings about transformation always takes place simultaneously with the invention of literature.” ( 31, on page 61) He sums up the parallel walk of two institutions in a chiasm which follows the claims about them not being a separate defined regions of being, but “'possibility of all that is made of letters' that bursts forth explosively with the 'things made of letters'” and “the possibility of all that is made of humans, that bursts forth explosively with the human thing”:

History is true history because it is a true novel – likewise, a novel is a true novel because it is true history, just as true history becomes fiction and fiction becomes history. The one always provokes the spontaneity of the realism of the other; the one is always non-knowledge, the inexpressible, impossible, unthinkable and inexperiencable, embedded in the expressible, possible, thinkable and experienceable of the other. ( 31 on page 62)

If the “autonomy of history is an illusion”, because “particular objects that it produces exist only in the language that history shares with other objects and productions” ( 31, on page 67), then the collapse of the distance between the two institutions and the drowning of history in general indistinguishability from heroically conceived literature is an equally problematic notion that equally blinds us to the specific “poetics of knowledge” ( 13) of history, its tropological fabric, and the literariness in its background.

Therefore, this text should by no means be read as a colonial gesture from a literary perspective, in which history is portrayed as an inferior ancilla, a less capable cousin, or merely a subspecies of literature. Rather, the aim was to explore how different ways of conceptualizing the politics of literature can help us examine the literariness of history and the already recognized political implications of its recognition, but nonetheless attend to the differences between concrete discursive embodiments of literariness “conceived at such a broad level of generality” ( 10 on page 79). This perspective is undoubtedly partial and could be well complemented by a complementary inquiry that draws attention to the mechanisms of history writing that tame the work of literariness so broadly conceived, that is, the radical democracy of writing in various other discourses, including (modern) literature, or by an inquiry that shifts the focus from an analytical emphasis on textual mechanisms to an institutional and broader social context. However, these potentially equalizing investigations are beyond the competences of a literary theorist and, thus, beyond the scope of this paper.

## Ethics and consent

Ethical approval and consent were not required.

## Data Availability

No data associated with this article.
