# Supplementary material for: First as a Tragedy, Then as some Other Figure: On Tropology of History and Politics of Literature
Source: Open Res Eur. 2025 Jul 25;5:194. [Version 1] doi: 10.12688/openreseurope.20776.1 (PMC12784047; doi:10.12688/openreseurope.20776.1)
Supplement: Peer review history

# Reviewer report: Reviewer response for version 1

**DOI:** 10.21956/openreseurope.22479.r63739

**Published:** 2026-01-12

**Authors:** Sara Raimondi

**Affiliations:** 1 Northeastern University London, London, UK

This article offers an ambitious and original synthesis of Hayden White’s tropological analysis of historical discourse with post-foundational political theory, particularly the work of Ernesto Laclau, Jacques Rancière, and Oliver Marchart. The central argument establishes a structural analogy between White’s claim that historical narratives are tropologically constructed and post-foundational ontology’s insistence on the absent-yet-necessary ground of the social. The article then explores whether various conceptions of “the politics of literature” can illuminate the political stakes of historiographical practice, examining modernist narrative strategies as potential interventions. Ultimately, the author advocates a nuanced engagement that recognises shared theoretical premises while respecting disciplinary specificities of the fields engaged. 

 The work maintains strong elements of originality in its demonstrating that White’s tropology and post-foundational political thought share fundamental epistemological and ontological commitments that have not been adequately recognised in existing scholarship. The parallel drawn between White’s “metaphysical principle” (p.3 of 16)—whereby similarity is ascribed onto singular and disparate historical events and phenomena—and Laclau's hegemonic articulation of antagonism that permeate the field of the social (p.4 of 16) is particularly insightful. The extension of this insight through Marchart's taxonomy of post-foundationalism provides a sophisticated framework for understanding White’s controversial position within historiography by putting it in dialogue with a different field of study. The engagement with Rancière also adds value and helps the author advance the point that generalised literariness and modernist form do not automatically produce political emancipation: this shows the limitations of applying literary politics directly to historiography. More generally, the engagement with post-foundational theory is very solid. The article demonstrates command of Laclau's development from Hegemony and Socialist Strategy through The Rhetorical Foundations of Society, recognising how his framework becomes increasingly tropological over time. The treatment of Marchart's distinction between foundationalism, anti-foundationalism, and post-foundationalism provides essential conceptual scaffolding. 

 However, some further areas could also be expanded: first, the references to post-foundationalism hinge on works now dated a few decades, and it would be interesting to test whether the arguments can be extended and expanded to encompass more recent developments in this field of political thought. Other key references belonging to this field, such as Chantal Mouffe's development of Laclau's insights toward agonistic democracy would seem directly relevant—given the article’s concern with multiple competing narratives and the impossibility of neutral ground—but remain absent. The section of thought of the key authors discussed, such as Ranciere’s itself, is also selective, with some noticeable omissions: for instance, there is only marginal reference to the critique to consensus politics/police that is central to Ranciere’s work on politics and aesthetics and that, if mobilised here, could strengthen the argument about how realist historiography produces “docile subjects” (in footnotes, p.5 of 16). 

 From a structural point of view, some of the transitions between chapters appear abrupt and not sufficiently justified. For instance, when moving between Chapter II and III, the transition to literary politics could be made stronger by expanding on why this move matters for post-foundational political thought. The section on De Certeau remains partially isolated and does not develop sufficiently what comparison and what addition it brings to the previous discussion of post-foundationalism (and Laclau in particular).  

 Overall, the article makes a sophisticated and original theoretical contribution that significantly advances understanding of the political dimensions of historical representation. By so doing, it manages to successfully navigate an innovative dialogue between historiography, literary theory, and political philosophy without collapsing their distinctions—a notable achievement given its critique of uncritical interdisciplinarity.

If any, are all the source data and materials underlying the results available?

No source data required

Is the work original in terms of material and argument?

Yes

Is the work clearly and cogently presented?

Partly

Is the argument persuasive and supported by evidence?

Yes

Does the research article contribute to the cultural, historical, social understanding of the field?

Yes

Does it sufficiently engage with relevant methodologies and secondary literature on the topic?

Partly

Reviewer Expertise:

Political theory; political thought; continental philosophy; post-structuralism

I confirm that I have read this submission and believe that I have an appropriate level of expertise to confirm that it is of an acceptable scientific standard.

# Reviewer report: Reviewer response for version 1

**DOI:** 10.21956/openreseurope.22479.r65728

**Published:** 2026-01-08

**Authors:** Ralph J. Poole

**Affiliations:** 1 University of Salzburg, Salzburg, Austria

The article offers a theoretically dense contribution to debates at the intersection of literary theory, narratology, and the philosophy of history. While framed through Hayden White’s tropology of historical discourse, the argument is situated within problems central to literary studies: literariness, narrative form, modernism, polyphony, and the politics of representation. By placing White in dialogue with Laclau, Rancière, Bakhtin, LaCapra, and De Certeau, the author offers interdisciplinary perspectives while also cautioning against the uncritical collapse of disciplinary boundaries. Especially the engagement with literary modernism is convincing, and the reading of Michelet as a proto-modernist figure, while critically distancing itself from Rancière’s more celebratory account, is effective in illustrating how even highly experimental literary-historical forms remain subject to hegemonic fixation.

 Although the article draws extensively on literary theory, its primary argumentative horizon sometimes appears oriented more toward historiography than literary studies proper. (Several footnotes, for example, contain arguments of central relevance to literary theory that could be integrated into the main text to ensure a more literature-oriented perspective, which literary scholars like myself may struggle to identify the article’s specific contribution to their field.) An analysis of one explicitly literary case (such as a modernist novel or political novel) would help to demonstrate how the theoretical framework operates when literature is not merely a model but the primary object. Stylistically, the article is written in a dense, sophisticated, and precise manner. I wonder, though, if the questions ending each section are meant rhetorically or have been answered in the end. Also, while I praise the intense engagement with this illustrious group of theorists, I would have liked an update on what is at stake for literary studies today, e.g. when viewed through the lens of feminist or postcolonial historiography.

If any, are all the source data and materials underlying the results available?

No source data required

Is the work original in terms of material and argument?

Yes

Is the work clearly and cogently presented?

Yes

Is the argument persuasive and supported by evidence?

Yes

Does the research article contribute to the cultural, historical, social understanding of the field?

Yes

Does it sufficiently engage with relevant methodologies and secondary literature on the topic?

Partly

Reviewer Expertise:

literary studies, cultural theory, gender studies

I confirm that I have read this submission and believe that I have an appropriate level of expertise to confirm that it is of an acceptable scientific standard.

# Reviewer report: Reviewer response for version 1

**DOI:** 10.21956/openreseurope.22479.r61307

**Published:** 2025-10-10

**Authors:** Kristiawan Indriyanto

**Affiliations:** 1 Universitas Prima Indonesia, Medan, Indonesia

Overall, this is a well written and well researched paper in which the argument is presented in sufficient details. There is only some minor issues in which I would like the writer to further expand in detail.

 While overall the dialectics of discussion is well presented, and I can see the overreaching structure, some parts seems like claim instead of an argument. For example 

 In the ending of the second chapter 

 Contemporary discussions of the politics of literature resonate with analogous themes and revolve around the questions that White raises within the historical field, especially in his later works (there is no reference to support this claim, in which readers, who might be unfamiliar to this topic do not know what exactly is the recent discussion of this topic)

 similarly on the next chapter, the second sentence

**For a long time**, this topic has mainly been viewed through the prism of the engaged literature.  (how long exactly, and what is the relation with the tropes of engaged literature)

 Lastly, the ending of your article seems abrupt. You suddenly introduce a new idea that the next inquiry on this topic is 'beyond the scope of this paper' and then the paper ends suddenly. 

 Perhaps you may revise the ending, (conclusion) I might say.

If any, are all the source data and materials underlying the results available?

Yes

Is the work original in terms of material and argument?

Yes

Is the work clearly and cogently presented?

Yes

Is the argument persuasive and supported by evidence?

Yes

Does the research article contribute to the cultural, historical, social understanding of the field?

Yes

Does it sufficiently engage with relevant methodologies and secondary literature on the topic?

Partly

Reviewer Expertise:

Literature

I confirm that I have read this submission and believe that I have an appropriate level of expertise to confirm that it is of an acceptable scientific standard.
